# Social Determinants of Health in Aging Populations: A Comparative Study of U.S.-Born and Immigrant Individuals

**DOI:** 10.1093/geroni/igaf122.265

**Published:** 2025-12-31

**Authors:** Man Guo, Yi Wang

**Affiliations:** University of Iowa, Iowa City, Iowa, United States; University of Iowa School of Social Work, Iowa City, Iowa, United States

## Abstract

Aging experiences and health outcomes are shaped by multi-dimensional social determinants of health (SDOH), yet the latent structures of SDOH and their health impacts remain underexplored, particularly among diverse older populations. Using 2016/2018 Health and Retirement Study data, this study employs multi-group confirmatory factor analysis (MG-CFA) to uncover SDOH structures and their links to ADL limitations, depression, and cognition among U.S.-Born (N = 9,258) and Immigrant adults aged 50 + (N = 1,379). MG-CFA (metric model: CFI = 0.966, RMSEA = 0.059) identified three consistent factors—Neighborhood Environment Quality (high cohesion, low disorder), Personal Resource Capacity (SES), and Family Support (child/family support)—with uniform measurement across groups. However, group-specific factor intercepts varied: U.S.-born adults reported higher SES and neighborhood cohesion, while immigrants experienced more neighborhood disorder but stronger family support, reflecting nativity-specific challenges and strengths. Regression analyses revealed that Personal Resource Capacity was robustly associated with better health outcomes across both groups, while Family Support was linked to fewer ADL limitations and depressive symptoms. Neighborhood Environment Quality showed limited direct health associations, suggesting its influence may be distal or mediated by more proximal resources. Non-significant interactions between SDOH factors and nativity status indicate that these determinants similarly benefit both groups. These findings advance gerontological theory by demonstrating uniform SDOH structures and health associations across nativity status despite baseline disparities. They also highlight the need for targeted interventions to address immigrant-specific barriers, such as education gaps and neighborhood disadvantage, while leveraging cultural strengths like family support to promote equitable aging outcomes.

